# Exploring New Tools in Upper Limb Rehabilitation After Stroke Using an Exoskeletal Aid: A Pilot Randomized Control Study

**DOI:** 10.3390/healthcare13010091

**Published:** 2025-01-06

**Authors:** Pantelis Syringas, Vassiliki Potsika, Nikolaos Tachos, Athanasios Pardalis, Christoforos Papaioannou, Alexandros Mitsis, Emilios E. Pakos, Orestis N. Zestas, Georgios Papagiannis, Athanasios Triantafyllou, Nikolaos D. Tselikas, Konstantina G. Yiannopoulou, George Papathanasiou, George Georgoudis, Daphne Bakalidou, Maria Kyriakidou, Panagiotis Gkrilias, Ioannis Kakkos, George K. Matsopoulos, Dimitrios I. Fotiadis

**Affiliations:** 1 Biomedical Engineering Laboratory, National Technical University of Athens, 9, Herοon Polytechniou Str., Zografou, 15773 Athens, Greece; ikakkos@biomed.ntua.gr (I.K.); gmatsopoulos@biomed.ntua.gr (G.K.M.); 2 Unit of Medical Technology and Intelligent Information Systems, Department of Materials Science and Engineering, University of Ioannina, 45110 Ioannina, Greece; vpotsika@uoi.gr (V.P.); ntachos@gmail.com (N.T.); pardalisathanasios@gmail.com (A.P.); chpapaioannou@cs.uoi.gr (C.P.); alexmitsis07@gmail.com (A.M.); fotiadis@uoi.gr (D.I.F.); 3 Laboratory of Orthopaedics and Biomechanics, Department of Orthopaedics, Medical School, University of Ioannina, 45110 Ioannina, Greece; epakos@uoi.gr; 4 CNA Lab, Department of Informatics and Telecommunications, University of Peloponnese, 22100 Tripoli, Greece; o.zestas@go.uop.gr (O.N.Z.); ntsel@uop.gr (N.D.T.); 5 Biomechanics Laboratory, Physiotherapy Department, University of the Peloponnese, 23100 Sparta, Greece; grpapagiannis@yahoo.gr (G.P.); athanat@gmail.com (A.T.); m.kyriakidou@uop.gr (M.K.); p.gkrilias@uop.gr (P.G.); 6 Physioloft, Physiotherapy Center, 14562 Kifisia, Greece; 7 Neurological Department, Henry Dunant Hospital Center, 11526 Athens, Greece; k.giannopoulou.14@hotmail.com; 8 Laboratory of Neuromuscular and Cardiovascular Study of Motion, Physiotherapy Department, Faculty of Health and Care Sciences, University of West Attica, 12243 Egaleo, Greece; gpapa@uniwa.gr (G.P.); dafbak@otenet.gr (D.B.); 9 Research Laboratory of Musculoskeletal Physiotherapy, University of West Attica, 12243 Athens, Greece; ggeorge@uniwa.gr; 10 Biomedical Research Institute, Foundation for Research and Technology-Hellas (FORTH), 70013 Heraklion, Greece

**Keywords:** rehabilitation, stroke, spasticity, robotic exoskeleton hand, soft glove, augmented reality games, upper limb

## Abstract

Background/Objectives: Spasticity commonly occurs in individuals after experiencing a stroke, impairing their hand function and limiting activities of daily living (ADLs). In this paper, we introduce an exoskeletal aid, combined with a set of augmented reality (AR) games consisting of the Rehabotics rehabilitation solution, designed for individuals with upper limb spasticity following stroke. Methods: Our study, involving 60 post-stroke patients (mean ± SD age: 70.97  ±  4.89 years), demonstrates significant improvements in Ashworth Scale (AS) scores and Box and Block test (BBT) scores when the Rehabotics solution is employed. Results: The intervention group showed slightly greater improvement compared to the control group in terms of the AS (−0.23, with a confidence interval of −0.53 to 0.07) and BBT (1.67, with a confidence interval of 1.18 to 2.16). Additionally, the Rehabotics solution was particularly effective for patients with more severe deficits. Patients with an AS score of 3 showed more substantial improvements, with their AS scores increasing by −1.17 ± 0.39 and BBT scores increasing by −4.83 ± 0.72. Conclusions: These findings underscore the potential of wearable hand robotics in enhancing stroke survivors’ hand rehabilitation, emphasizing the need for further investigations into its broader applications.

## 1. Introduction

Stroke stands as a prominent contributor to enduring disability on a worldwide scale, often leading to the development of upper limb spasticity [1]. Spasticity manifests through heightened muscle tension, stiffness, exaggerated tendon reflexes, involuntary muscle contractions, and a resistance to passive limb movement [2]. In a recent study, stroke incidents worldwide were recorded at 12.2 million, with 101 million existing cases, 6.55 million deaths from stroke, and 143 million disability-adjusted life years (DALYs) [3]. Upper limb impairment is the most common disability and affects 77.4% of patients [4]. Advancements in the application of robotic devices have demonstrated potential in supporting the restoration of hand function [5].

Efficient rehabilitation is crucial for restoring functionality and enhancing the quality of life (QoL) for stroke survivors [6]. The ability to regain dexterity and functionality in the hand is crucial for daily activities and QoL [7]. In the pursuit of improving motor functions, exoskeleton hand rehabilitation robots have been commonly utilized [8]. They imitate the structure of human limbs, attaching to different points on the patient’s body and aligning their joint axes with those of human joints. Additionally, they can be securely fastened to a table or possess mobility options, either being mobile or stationary relative to the patient’s body [9].

Exoskeletons are created as wearable electromechanical devices aimed at augmenting the physical capabilities of the person wearing them [10,11,12,13]. The literature lacks a definitive consensus regarding the superiority of robotic devices over conventional physiotherapy (CP) [14]. While robotic devices offer the advantage of optimizing therapists’ time, it remains unclear whether they yield superior rehabilitation outcomes compared to CP [15]. Some studies have suggested that the repetitive training of isolated movements and robot-assisted training may have a more significant impact on stroke-related motor impairments than simply increasing therapy duration alone [16].

Conventional physiotherapy (CP), although indispensable for the recovery process, frequently encounters obstacles such as limited patient engagement and a lack of personalization. Notably, serious games have been proven to be highly effective in enhancing patient motivation [17]. Concurrently, the integration of innovative technologies, such as robotic exoskeletons and augmented reality (AR), has exhibited promising outcomes in the rehabilitation of patients with motor impairments [18].

The concept of employing serious games in the domain of rehabilitation is not a recent development. In as early as 2009, Burke et al. established the fundamental principles of game design suitable for upper limb stroke rehabilitation, resulting in the creation of several games rooted in these principles [17]. Notably, the challenge of customizing games for rehabilitation purposes, as elucidated in [19], primarily arises from the intricacies inherent in rehabilitation, often necessitating human intervention. For instance, research on game usability has encompassed comprehensive assessments involving interviews with both rehabilitation professionals and their patients [20]. An intriguing proposal by Rabin et al. [21] involved an investigation into the impact of serious games on motor control through the development of a progressive upper-limb rehabilitation game.

Taking the factors mentioned above into consideration, these challenges underscore the necessity for creative solutions that can provide support to both therapists and patients throughout the rehabilitation process in both clinical environments and at home [17]. A lightweight, wearable, and low-cost exoskeleton may allow patients to continue daily therapy at home or to serve as an assistive device for activities of daily living (ADLs), reducing physical strain on therapists while enabling more rigorous and prolonged training sessions for patients [22,23]. Many robotic systems are focused on improving gross reaching movements of the upper extremity [24].

Among the commonly used metrics, the Ashworth Scale (AS) and Box and Block test (BBT) are widely recognized as reliable indicators of motor function and dexterity [25,26]. These metrics provide quantitative measures that reflect patients’ ability to perform daily tasks, thereby serving as proxies for functional improvement and overall QoL. By evaluating rehabilitation outcomes using these scores, clinicians and researchers can assess the efficacy of therapeutic interventions and their real-world impact on patients’ lives.

This paper aims to evaluate the effectiveness of the Rehabotics solution, a system that delivers physiotherapy through the integration of AR serious games and an exoskeleton glove, compared to conventional physiotherapy. Improvements in upper limb function, as assessed using the AS and BBT, will serve as primary outcomes. The study employs a randomized clinical trial design.

## 2. Materials and Methods

In previous work, the authors described the architecture of Rehabotics, outlining its key functionalities, such as AS and BBT assessment, functional and stretching exercises, Rehabilitation AR serious games, data logging, monitoring, and analysis [27,28]. Additionally, one study explored the use of AR BBT as an alternative quantitative assessment method [29].

### 2.1. The Rehabotics Exoskeletal Aid

The Rehabotics exoskeletal aid has been developed based on two fundamental components: (i) the Passive Exoskeletal Aid (PEA) and (ii) the Active Exoskeletal Aid (AEA), aiming to support healthcare professionals in assessing and personalizing upper extremity function in patients with post-stroke spasticity. This study focuses on AEA; however, the PEA is also briefly mentioned, as it forms an integral part of the Rehabotics system.

The PEA is a soft glove equipped with an array of integrated sensors, including the technology of CaptoGlove© [30]. Among these sensors are individual bending sensors on each finger, allowing for the independent measurement of finger bending and pressure sensors located on the fingertips to gauge applied pressure levels [27].

**Figure 1:**
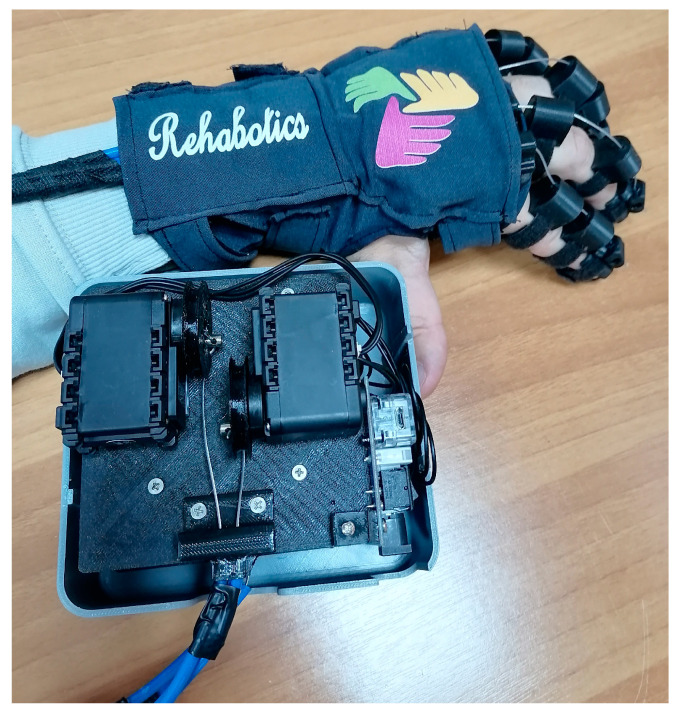
The Rehabotics exoskeletal aid with actuators.

The role of the AEA is also crucial, as stretching training exercises represent a fundamental component of a comprehensive rehabilitation program [24]. The AEA has been developed as an appropriate application to fully manage finger movements through the rotational motion of servo motors (Dynamixel AX-12A, Corona, CA, USA). This device is characterized as an articulated structure comprising specially designed rings through which a wire is guided, responsible for transferring the kinetic energy of a servo motor to the finger structures (converting rotational motion into linear). These sections have a crescent shape for automatic alignment with the user’s finger. Each finger has three Velcro straps on its three bones, which can be adjusted to fit any finger size. To stabilize the exoskeletal aid on the patient’s hand, stabilizers have been adapted at two points. One point is the patient’s wrist, which can be adjusted to fit any wrist type through a structure (Figure 1).

**Figure 2:**
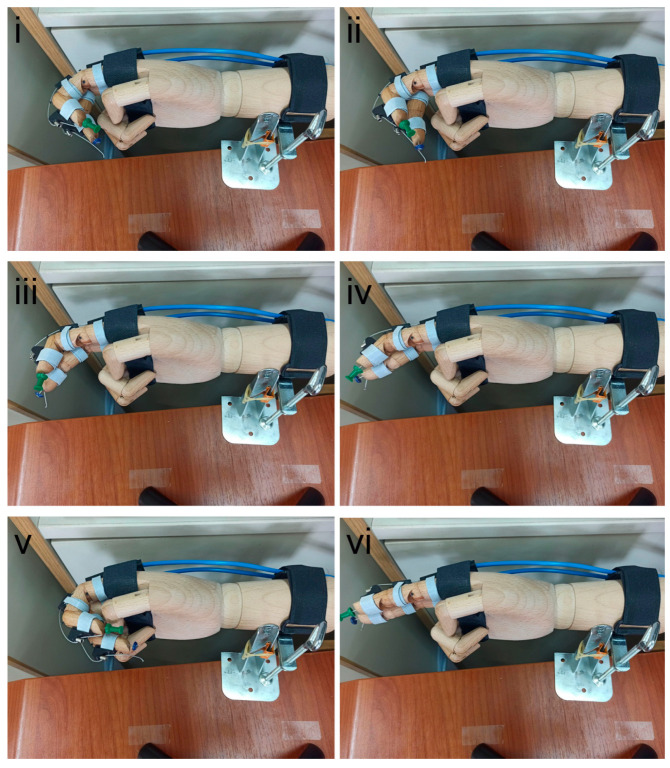
Finger (index and middle) movement. (**i**) Initial clenched hand position, (**ii**–**iv**) progressive stages of finger extension as the actuators gradually stretch the index and middle fingers, (**v**) return to the starting clenched position, and (**vi**) re-initiation of the extension cycle.

An image showing the movement of two fingers is presented below (Figure 2). The AEA utilizes actuators to gradually stretch the index and middle fingers, transitioning from a clenched hand position (i) through intermediate stages (ii to iv), then returning to the starting position (v) before initiating another extension (vi).

Furthermore, it provides an interactive interface for initiating or terminating exercises and allows the physical therapists to input and modify parameters, such as the finger extension speed, define the maximum extension angle, and specify the speed of return to the initial position. Through a user-friendly navigation menu, the physical therapist initiates the exercise, and the servo motors begin finger movements. Upon completion of all repetitions, the angles of movement for each servo motor are displayed in degrees on the graphical interface. Furthermore, there is an option for assisted finger retraction by the servo motor.

### 2.2. AR Serious Games Platform

The notion of “serious games” pertains to the development of interactive tools designed to facilitate the rehabilitation process, simultaneously fostering engagement and bolstering motivation. These serious games serve a dual role in rehabilitation. Initially, they reinvigorate CP exercises, effectively transforming them into captivating and enjoyable experiences, thereby elevating patient motivation. Secondly, the games function as objective, quantitative instruments for evaluating the patient’s progress.

Evaluating hand motor functions through ADLs represents a holistic approach to assessing an individual’s upper limb capabilities in real-life contexts. There are several tests based on ADL, most created decades ago but still extensively used, such as the Jebsen Hand Function Test [31] and the Sollerman Hand Function Test (SHT) [32]. This comprehensive methodology involves analyzing how an individual performs a range of everyday tasks that require manual dexterity, fine motor control, and hand-eye coordination. These activities may include gripping and manipulating objects, buttoning clothes, brushing teeth, self-feeding, and more. The majority of activities featured in assessment tests centered around ADLs can also be used as repetitive hand rehabilitation exercises.

In [33], as part of the Rehabotics solution, a rehabilitation assessment suite was implemented based on the SHT. This system eliminates the need for specialized equipment or high computational resources, requiring only a mid-range PC and a standard camera. The AR serious game employs advanced computer vision and image recognition algorithms, bundled into a lightweight standalone application. Its high-level architecture integrates OpenCV for image processing and MediaPipe Hands [34] for efficient and accurate hand tracking.

The software design involves three key stages: modeling the computer vision environment, detecting and tracking hand movements, and applying the operational logic to captured frames. The virtual environment is created using a single RGB camera, capturing 60 frames per second.

**Figure 3:**
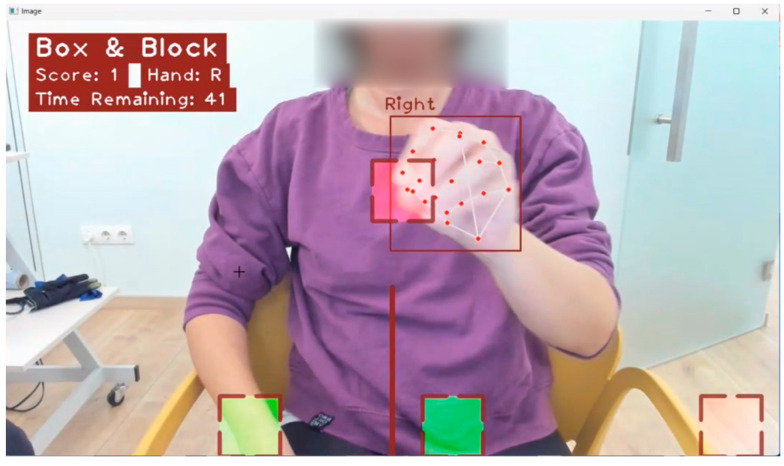
AR BBT. The squares represent the virtual blocks, which the patient interacts with and moves during the task.

Hand detection and operation handling are powered by MediaPipe’s ML pipeline, which uses a palm detector and a hand landmark model to provide high-quality 2.5D landmarks. This setup allows real-time tracking using a low amount of resources. The software identifies hand poses and the different hand grips, calculating the distances between the fingers. The system calculates the position of the hand relative to the camera and ensures that the participant interacts realistically with the environment. The AR serious games platform integrates a collection of “Sollerman” games, such as Zip/Unzip Wallet, Key Lock, Open Jar, and Use Screwdriver and Draw, with some of these detailed in [33,35,36], and other games such as Balloon Pop and Rock-Paper-Scissors, along with the Box and Block test (BBT) [37]. A photo from AR BBT is presented in Figure 3.

The SHT is specifically designed to provide an accurate assessment of overall hand function, focusing solely on hand grips rather than elbow or shoulder movements. Its primary purpose is to capture a realistic picture of grip functionality in ADLs and to reflect the most commonly used hand grips in everyday tasks. According to Sollerman, there are eight main hand grips utilized in ADLs, with pulp pinch, lateral pinch, five-finger pinch, and diagonal volar grip being the most dominant [32].

In this study, six AR serious games were employed to target and improve these fundamental grips. The BBT, Zip/Unzip Wallet, and Balloon Pop games engaged the pulp pinch, the Key Lock game focused on the lateral pinch, the Open Jar game enhanced the five-finger pinch, and the Use Screwdriver game targeted the diagonal volar grip. Notably, four of these games (Zip/Unzip Wallet, Key Lock, Open Jar, and Use Screwdriver) are also components of the SHT, as they simulate common ADLs, further aligning with the intervention with real-world functional tasks. Specifically, in the Zip/Unzip Wallet game, patients simulate zipping and unzipping a virtual wallet. The Key Lock game requires participants to turn a door lock key 90°. In the Open Jar game, the task involves removing a jar lid, while the Use Screwdriver game challenges participants to turn a screw using a virtual screwdriver.

All Sollerman games, except Balloon Pop, are evaluated using the Sollerman Hand Function Score. In the Balloon Pop game, patients receive 10 points for each balloon successfully popped. The BBT is scored on a scale from 0 to 150, with patients earning 1 point for each block successfully moved to the opposite compartment. Although the patients’ performance was evaluated after each game, we treated the serious games as rehabilitation exercises and did not analyze individual performance outcomes for the games in this clinical study.

### 2.3. Clinical Assessments

To evaluate the effectiveness of the intervention, two clinical assessments were employed: the AS and BBT.

The AS is one of the primary tools for assessing spasticity in individuals with mobility issues in the upper extremities undergoing rehabilitation [38]. The AS is measured on a score ranging between 0 and 4, with a higher score indicating increased difficulty in finger motion, while lower scores correspond to ease of finger motion [25]. More specifically, the clinical interpretation of the AS scores is presented below:

Score 0: No increase in muscle tone.

Score 1: Slight increase in muscle tone, with a catch and release or minimal resistance at the end of the range of motion when an affected part(s) is moved in flexion or extension.

Score 2: A marked increase in muscle tone throughout most of the range of motion, but affected part(s) are still easily moved.

Score 3: Considerable increase in muscle tone; passive movement is difficult.

Score 4: Affected part(s) rigid in flexion or extension.

The BBT consists of a wooden box divided into two compartments by a partition and includes 150 blocks. During the BBT administration, the patient is instructed to move as many blocks as possible, one by one, from one compartment to the other within 60 s. The higher the number of blocks moved, the better the patient’s manual dexterity.

**Figure 4:**
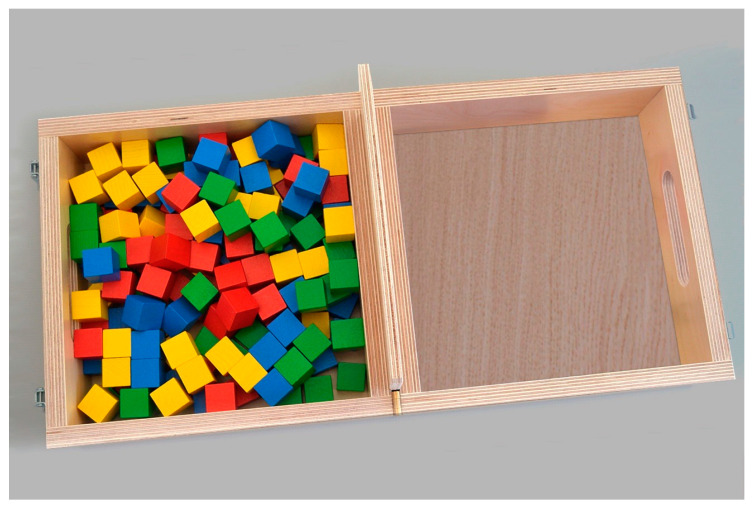
Conventional BBT.

Both assessments were conducted at baseline (T0), one week prior to the intervention, and at the end of the five-week intervention period (T1) to evaluate changes in spasticity and hand functionality over time. A photo from the Conventional BBT is presented in Figure 4.

### 2.4. Study Design

This section presents the design of the clinical study performed to assess the outcomes achieved using the Rehabotics solution compared to CP routines. A validation study was conducted at the Physioloft Physiotherapy Center (14562 Kifissia, Athens, Greece), consisting of the evaluation of Rehabotics by two clinicians to assess the performance and suitability of the exoskeletal aid as a means of providing therapy to stroke patients.

The inclusion criteria included stroke pathology, an AS ≤ 3, and a maximum of 6 months since the incident. The exclusion criteria were the presence of neurological pathologies that affected hand motion (Parkinson’s disease) or musculoskeletal deficits that affected the normal finger range of motion (e.g., finger fractures, joint stiffness/pain, arthritis that affected joint motion), AS > 3, and over 6 months since the incident.

The study was conducted in accordance with the Declaration of Helsinki and approved by the Ethics Committee of the University of Peloponnese on 8 September 2022 (No: 20366) and was registered with ClinicalTrials.gov (NCT06519630) on 25 July 2024. Informed consent was signed and obtained from all participants in the clinical study.

After initial assessment for eligibility, independent simple randomization was used to allocate participants to either the intervention group or the control group. The randomization was performed by a generated random number sequence with a 1:1 allocation ratio. The study was open-label because assessors and the participants were not blinded during the entire study period.

All participants underwent assessments conducted by two clinicians one week prior to the commencement of the study, referred to as the baseline evaluation (T0). Subsequently, after 5 weeks (T1), the participants were reassessed. The control group received CP, while the intervention group received physiotherapy administered by the Rehabotics solution. The clinicians performed two clinical assessments for AS and BBT for the patient’s affected upper limb. Both groups were assessed with the traditional AS and BBT. All participants completed the full five weeks of training, with no dropouts reported during the study period.

### 2.5. Rehabilitation Interventions

The rehabilitation sessions for participants in the intervention group were composed of a single repetition of each of the four Sollerman Games (Wallet, Key, Jar, Screwdriver), one repetition of Balloon Pop, and one repetition of AR BBT under controlled conditions. They were seated in ergonomic chairs and faced a fixed webcam positioned at 50 cm, aligned perpendicularly to them. Furthermore, to optimize the tracking system’s performance, the room was well lit. A clinician remained present throughout the session to provide assistance if patients encountered any difficulties. The clinician subsequently initiated a stretching program using the Active Exoskeletal Aid, conducting three sets of ten repetitions on the finger flexors of the patient’s hand. The rehabilitation sessions totaled 30 min in duration and were repeated for 3 days/week.

Respectively, a similar hand functional training program prescribed for the control group consisted of sessions lasting 30 min/day for 3 days/week. The control group’s hand functional and stretching training program aimed to enhance upper limb strength, dexterity, and coordination through repetitive, task-oriented exercises. Each session began with functional strength exercises to improve hand function. Repetitive, task-oriented tasks were used to simulate the ADLs, targeting the eight main grips defined by Sollerman [32]. The tasks ranged from simple daily activities, such as buttoning clothes and picking up small objects and utensils, to more complex ones, such as writing. The stretching program was implemented by an experienced and specialized physiotherapist and included stretching exercises for the flexor muscles of the fingers. Each stretch was held for 10 s, stopping just before triggering a myotatic reflex. Under the clinician’s supervision, proper technique was ensured, with adaptations made to accommodate the individual capabilities of each participant, ensuring both effectiveness and safety.

Both programs lasted for 5 weeks and were designed by two clinical experts.

### 2.6. Statistical Analysis

An a priori power analysis was performed using G*Power 3.1.9.7 software for Windows (Heinrich-Heine-Universität Düsseldorf, Düsseldorf, Germany) to determine the appropriate sample size required to achieve a large effect size with 80% power and a significance level of 0.05, employing an effect size index of 0.75. A non-directional (two-tailed) analysis was applied. The calculated sample size was 29 per group.

Demographic and clinical characteristics were summarized using means and standard deviations. To ensure groups were balanced in terms of participant characteristics, independent sample *t*-tests were used in age, stroke onset, and AS.

The comparison of clinical characteristics at pre-treatment (T0) and post-treatment (T1) was performed with the use of paired two-tailed *t*-tests for AS and BBT scores. AS and BBT scores were presented as absolute values and mean differences ± standard deviations.

A 2 × 2 repeated measures ANOVA test, with each group (the intervention group and the control group) as the between factor and time (two levels: pre-intervention and post-intervention) as the within factor, was used to compare the effectiveness of the physiotherapy methods. The treatment effects were compared by computing the mean difference with 95% confidence intervals between the two groups. The between-group effect size was determined utilizing Cohen’s d coefficient. A magnitude exceeding 0.8 denoted a large effect, 0.5 indicated a moderate effect, and less than 0.2 represented a small effect.

Finally, a prospective response analysis was conducted by categorizing the participants of the intervention group according to their T0 AS and running paired two-tailed *t*-tests within each subgroup to explore the Rehabotics effectiveness within distinct cohorts. The data were expressed as mean differences ± standard deviations.

## 3. Results

**Figure 5:**
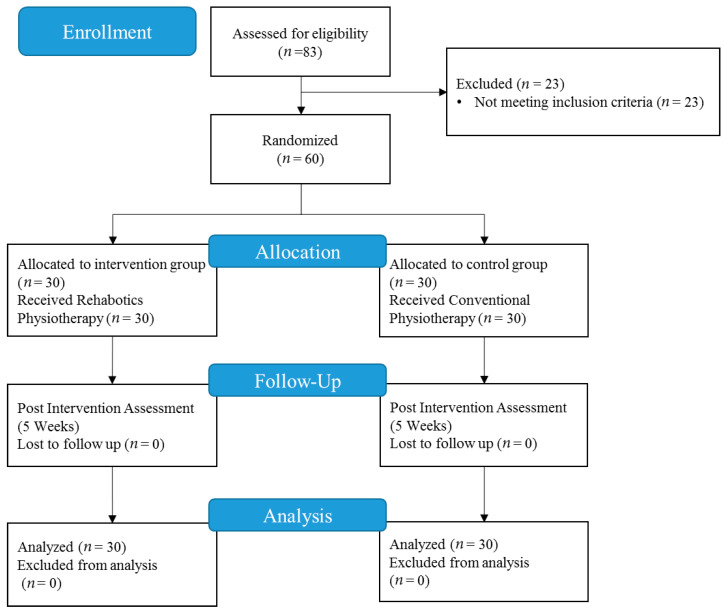
Flow diagram of the RCT.

A total of 83 patients were screened; 23 individuals were excluded prior to randomization, and 60 were randomized. The pilot study included 60 post-stroke patients (Figure 5), with an average age of 70.97 ± 4.89 years. Participants were randomly assigned to either the intervention group (*n* = 30) or the control group (*n* = 30). The demographic characteristics of the participants are presented in Table 1, demonstrating the two groups in terms of age, gender, stroke side, post-stroke months, and AS baseline. The t-student tests showed no significant differences in terms of age (*p* = 0.53), AS (*p* = 0.85), BBT (*p* = 0.71), and stroke onset (*p* = 0.38). The distribution of patients according to the baseline AS is also shown in Table 1.

### 3.1. Ashworth Scale

**Figure 6:**
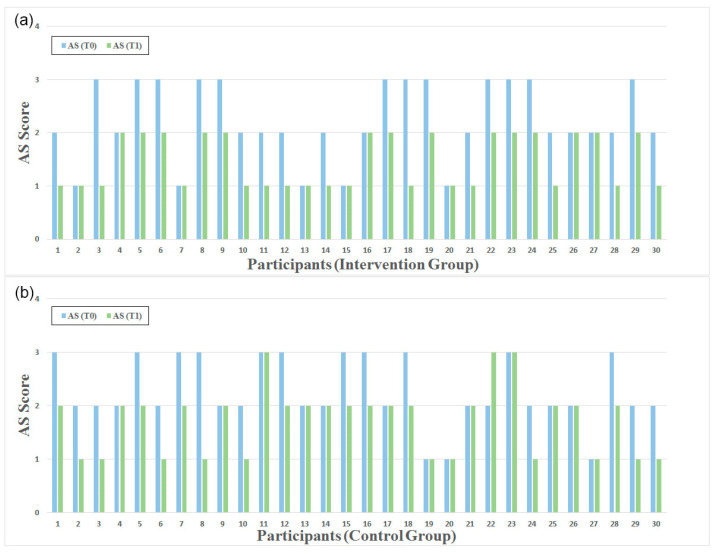
AS for participants in (**a**) intervention group and (**b**) control group at T0 (blue) and T1 (green).

The detailed results per participant and group are presented below in Figure 6. As shown in Table 2, we observed a change in the AS scores from 2.23 ± 0.73 at the pre-intervention assessment to 1.47 ± 0.51 at the post-intervention assessment in the participants of the intervention group. Meanwhile, the change in the control group was from 2.27  ±  0.64 at the pre-intervention assessment to 1.73 ± 0.64 at the post-intervention assessment. A notable decrease in muscle spasticity of the hands, as measured with the use of AS, was observed in both groups following the treatment compared to the pre-intervention (*p* < 0.05), evidenced by the reduction in AS scores.

Additionally, as can be seen in Figure 6, in the intervention group, two participants with a baseline of AS = 3 improved by 2 points. Also, one participant from the control group experienced a similar change. Furthermore, 19 patients in the intervention group improved by 1 point, while the rest maintained their initial AS. On the other hand, in the control group, 15 patients improved by 1 point, while 1 (patient 22) revealed a higher AS score compared to T0.

The intervention group showed slightly greater improvement compared to the control group in terms of AS scores: −0.23 (−0.53 to 0.07), although this difference was not statistically significant (*p* = 0.302). The effect size between the groups was moderate (Cohen’s d = −0.38). These results suggest there were no differences between the two approaches and the outcomes were equivalent.

### 3.2. BBT Score

**Figure 7:**
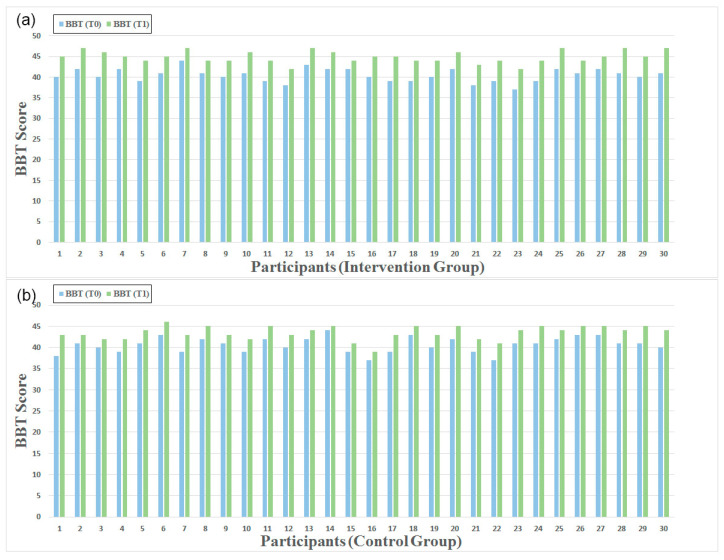
BBT score for participants in (**a**) intervention group and (**b**) control group at T0 (blue) and T1 (green).

After the 5-week intervention, we observed noticeable improvements in hand functionality for both groups, as depicted in Figure 7. The BBT score of the intervention group increased from 40.47 ± 1.61 to 45 ± 1.39. Notably, the control group also demonstrated significant changes, with a BBT score ranging from 40.63 ± 1.81 to 43.5 ± 1.57. Both within-group differences were significant (*p* < 0.05).

Moreover, within the intervention group, five patients showed a notable improvement, moving six additional blocks. Respectively, only one patient in the control group demonstrated a significant increase, moving five extra blocks.

According to the statistical analysis, the intervention group showed greater improvement in BBT 1.67 (1.18 to 2.16), with a trend towards significance (*p* = 0.096), but the effect size between the groups was large (Cohen’s d = 1.72), suggesting a clinically meaningful difference.

### 3.3. Subgroup Analysis

The analysis revealed changes in the Ashworth scores of the intervention group over the 5-week intervention period, categorized by baseline AS levels, as shown in Table 3. Participants with a baseline AS of 3 (*n* = 12) demonstrated a significant improvement of −1.17 ± 0.39 points in AS, along with an increased ability to move blocks by 4.83 ± 0.72, while those with an AS of 2 (*n* = 13) exhibited an increase of −0.69 ± 0.48 points and 4.62 ± 1.12 extra blocks. Finally, participants with a baseline AS of 1 (*n* = 5) did not show an increase in AS but showed an increased ability to move blocks by 3.6 ± 1.14. All comparisons yielded statistically significant results (*p* < 0.05). The statistical analysis suggested that participants with more severe deficits showed better response to Rehabotics treatment.

## 4. Discussion

In this paper, we presented the physiotherapy effectiveness of the Rehabotics rehabilitation solution for patients with upper limb spasticity after stroke. The results of our pilot study, based on within-group comparisons, demonstrate that the Rehabotics solution enhances physiotherapy outcomes. Additionally, the within-groups test indicated that its effectiveness is comparable to that of CP.

The drawback of the AS for patients’ assessment is that it does not consider other factors that may affect hand function, such as weakness or lack of coordination, but it evaluates only the spasticity of the hand [25,39]. Therefore, it was used in conjunction with other assessment tools, such as BBT, to provide a more comprehensive evaluation of hand function in individuals with hand impairments.

Both rehabilitation treatments demonstrated statistically significant improvements, as indicated by paired *t*-tests, confirming that both rehabilitation therapies are effective in hand functionality restoration after stroke. Comparing the two therapies, in terms of AS changes, no statistically significant difference was observed (*p* = 0.302), with a moderate effect size (Cohen’s d = −0.38) suggesting comparable outcomes between the two methods in reducing spasticity. In contrast, the intervention group showed greater improvements in terms of the BBT, with a mean difference of 1.67 (1.18 to 2.16) and a trend towards statistical significance (*p* = 0.096). Notably, the effect size for the BBT (Cohen’s d = 1.72) was large, indicating a clinically meaningful improvement in upper limb function for the intervention group. Although the current sample size may have limited statistical power, the observed effect size points towards the potential advantage of Rehabotics over conventional methods in terms of BBT increase. Future studies with larger sample sizes are warranted to further investigate this promising finding.

The subgroup analysis suggests that participants with more severe deficits, as indicated by higher AS scores (AS = 3 and AS = 2), showed better response to the Rehabotics treatment. This indicates that the Rehabotics treatment may be particularly beneficial for patients with higher levels of spasticity, potentially offering a greater therapeutic advantage for those with more severe motor impairments. However, due to the relatively small sample size within subgroups, further research with larger samples is needed to confirm this observation.

According to similar clinical trials, our study provides evidence supporting the significant improvements of patients using AR serious games in conjunction with robotic gloves [40]. Furthermore, the combined use of rehabilitation gloves with AR serious games produces significant improvements over the use of CP in the upper limbs of stroke patients, regardless of the duration of the intervention in both the short and long term and whether they were combined with CP [41,42].

The integration of robotics, AR, and serious games is able to provide a more engaging and motivating rehabilitation experience for patients. To support continuous rehabilitation and patient monitoring, serious game systems enhanced by computer vision techniques can be effectively utilized in clinical or home-based settings. Rehabotics provides patients with a solution that can be used at their own convenience, ensuring that they feel comfortable, safe, and at ease during their recovery process, with the flexibility to perform exercises independently. Intensive movement therapy for upper limb rehabilitation often results in patient frustration during conventional rehabilitation [43]. The gamification of therapeutic exercises ensures patients remain active and motivated, while they can also be closely tracked throughout their rehabilitation exercises, increasing their clinical adherence, acceptance, and engagement [44,45]. While it is possible that baseline differences in motivation or engagement could influence outcomes in unsupervised or remote settings, the clinical supervision in our study helped minimize such variability.

Although our study utilized a preset of serious games for each session, these games can be tailored to meet the unique needs and impairments of individual patients. The same applies to the stretching program of the exoskeletal aid, which is fully configurable. Customization allows clinicians to design targeted interventions that address specific functional deficits after analyzing the needs of each patient. By aligning the game content and exercises with the patient’s particular rehabilitation goals, Rehabotics can provide a more personalized therapeutic experience. This makes it a versatile tool for addressing diverse rehabilitation needs and effectively creating a patient profile. The patient profile can then be updated for each subject, enabling them to challenge their previous performance. This progressive training, with increasing difficulty and intensity, has been shown to effectively improve motor recovery [46].

A patient only interacting with virtual objects may lead to a loss in the sense of reality; that is why robotic devices can balance this disadvantage by providing haptic feedback and generating a strong sense of involvement in the task. Additionally, when addressing cognitive impairments, AR serious games are effective and easy to understand, as they combine virtual objects with real-world environments, making exercises more intuitive. Furthermore, they can be enhanced with mental exercises designed to promote neuroplasticity.

Compared to CP, which places a burden on the physiotherapists, Rehabotics offers the advantage of delivering repetitive movements consistently and precisely without placing additional strain on physiotherapists. By automating the execution of these movements, the robot allows therapists to focus on other critical aspects of care, such as monitoring progress, adjusting treatment plans, and providing personalized support. This capacity for repetitive exercises has been shown to yield promising outcomes, enhancing patient recovery by promoting neuroplasticity and improving motor function over time [47].

Although the application of robotics is widely used in stroke rehabilitation, only a few clinical studies have been performed with a limited number of participants [48]. Only one study from S. M. Linder et al. surpassed ours in terms of participant enrollment, featuring 99 participants [49]. The existing literature on systems integrating serious games with robotic-assisted physiotherapy is particularly sparse, often lacking assessment tools in their setups [42,50].

There are several limitations to this clinical study. While the study provides valuable insights into the effectiveness of Rehabotics within clinical parameters, the absence of home-based testing may limit the generalization of the findings to real-world, day-to-day scenarios, where stroke survivors often seek to regain functional independence. It is important to note that there were no reported cases of discomfort associated with the use of the Rehabotics solution. Additionally, most participants exhibited the capability to independently put on the glove and expressed their willingness to utilize the device in their home environment. Future research should consider incorporating home-based assessments to provide a more comprehensive understanding of the Rehabotics’ practical utility and performance in a broader range of settings.

Furthermore, follow-up was absent from our clinical study, and it is necessary to assess the effectiveness of rehabilitation interventions. Studies indicate that recovery plateaus after approximately one and a half years after stroke [51]. Integrating a follow-up over 1 or 2 years might provide useful information in terms of long-term effectiveness, as well as the sustainability of the treatment. In future research studies, lengthening the period of rehabilitation past 5 weeks could make it possible for a much more extensive assessment of treatment efficiency and any necessary adjustments to maximize the treatment outcome.

In future studies, it would be valuable to further explore how the integration of serious gaming, as part of a rehabilitation program, compares to traditional robotic rehabilitation in terms of overall effectiveness, patient engagement, and functional outcomes. While our study focused on evaluating the Rehabotics system using specific clinical assessments, examining the comparative advantages of serious gaming versus conventional robotic therapies could provide deeper insights into their relative impacts on rehabilitation efficiency.

We believe the key contribution of this study is its comparison of conventional and robotic therapy, providing both an equivalent practice time while reducing the need for resources. Rehabotics enhances both the clinical applicability and effectiveness of robotic therapy. Demonstrating the economic sustainability of robotic therapy is essential for promoting its wider adoption by minimizing the additional costs associated with robotic devices.

## 5. Conclusions

This clinical study underscores the therapeutic potential of Rehabotics in stroke rehabilitation, demonstrating improved hand motor function through the use of robotics and AR games. The positive results obtained from the clinical study reinforce Rehabotics as an integrated system, serving as a rehabilitation tool, offering advanced interventions for individuals recovering from stroke.

## Figures and Tables

**Table 1:** Participant’s demographic and baseline data.

	Intervention	Control
Age Mean (SD)	71.37 (5.2)	70.57 (4.62)
Gender (Male/Female)	18/12	16/14
Stroke Side (Right/Left)	15/15	12/18
Post-Stroke Months Mean (SD)	4.49 (1.02)	4.73 (1.09)
AS Mean (SD)	2.23 (0.73)	2.27 (0.64)
AS = 3	12/30 (40.00%)	11/30 (36.67%)
AS = 2	13/30 (43.33%)	16/30 (53.33%)
AS = 1	5/30 (16.67%)	3/30 (10.00%)
AS = 0	0/30 (0%)	0/30 (0%)

**Table 2:** Changes in Outcome for Ashworth Score and BBT for within Groups.

	Intervention			Control			Difference Between Groups	
	T0	T1	Difference T1–T0	T0	T1	Difference T1–T0	Mean (95% CI)	*p*-Value
AS	2.23 (0.73)	1.47 (0.51)	−0.77 (0.57)	2.27 (0.64)	1.73 (0.64)	−0.53 (0.63)	−0.23 (−0.53 to 0.07)	0.302
BBT	40.47 (1.61)	45 (1.39)	4.53 (1.04)	40.63 (1.81)	43.5 (1.57)	2.87 (0.9)	1.67 (1.18 to 2.16)	0.096

**Table 3:** Changes in Outcome for Ashworth Score and BBT for within Intervention Subgroups.

AS Subgroup		T0	T1	Difference T1–T0	*p*-Value (t-Value) T1–T0
3 (*n* = 12)	AS	3 (0)	1.83 (0.39)	−1.17 (0.39)	6.07 × 10^−10^ (10.38)
	BBT	39.5 (1.09)	44.33 (1.44)	4.83 (0.72)	1.03 × 10^−10^ (11.41)
2 (*n* = 13)	AS	2 (0)	1.31 (0.48)	−0.69 (0.48)	2.53 × 10^−5^ (5.2)
	BBT	40.54 (1.45)	45.15 (1.46)	4.62 (1.12)	2.66 × 10^−8^ (8.08)
1 (*n* = 5)	AS	1 (0)	1 (0)	0 (0)	-
	BBT	42.6 (0.89)	46.2 (1.3)	3.6 (1.14)	9.4 × 10^−4^ (5.09)

## Data Availability

The data presented in this study are available upon request from P.S. (the first author). The data are not publicly available due to privacy reasons.
